# #Putkids1st: Health Professionals Using Social Media for Public Policy Advocacy—From Collective Action to Connective Action

**DOI:** 10.3390/children10081343

**Published:** 2023-08-03

**Authors:** Charles Wood, Pierangelo Rosati, Theo Lynn

**Affiliations:** 1 Collins College of Business, The University of Tulsa, Tulsa, OK 74104, USA; charles-wood@utulsa.edu; 2 J.E. Cairnes School of Business and Economics, University of Galway, H91 YK8V Galway, Ireland; 3 Irish Institute of Digital Business, Dublin City University, D09 RFK0 Dublin, Ireland; theo.lynn@dcu.ie

**Keywords:** pediatricians, electronic word of mouth, public policy advocacy, professional organizations, healthcare, #putkids1st

## Abstract

This study examines public policy advocacy by pediatricians and other health professionals in the hashtag community: #putkids1st. The study explores 4321 tweets that feature the hashtag, generated by 1231 unique users largely drawn from the American Association of Pediatricians and its members. The data are used to explore the structural dynamics of the hashtag community, the role of homophily, and to test a source-message framework to predict and recommendations to help improve engagement and retransmission of professional health advocacy messages.

## 1. Introduction

At least 250 million children worldwide were not able to reach their full physical or psychological development in 2016 [1]. In 2019, up to 1 billion were affected by abuse or neglect [1]. Ensuring children’s rights to survival, health, education, and protection has been a central component of international human rights treaties for over a century from the establishment of the League in Nations in 1924 to the United Nations Convention on the Rights of the Child in 1989, the most widely ratified UN human rights treaty [2]. Interestingly, the US signed the treaty but did not ratify it.

There is a long history of pediatric public health advocacy dating back to the 19th century [3,4]. This form of advocacy is defined as the use of information and resources to reduce the occurrence or severity of health problems that affect a substantial proportion of people in a community and is not confined to clinical settings [5]. As children have little political voice of their own, others are required to speak on their behalf. Indeed, the foundation of the American Academy of Pediatrics (AAP) was based on the advocacy position taken by a group of pediatricians in support of the first federal legislation to support pregnant women and new mothers [3]. Common pediatric advocacy activities include education, publications and media engagement, lobbying, testifying, and encouraging voter participation [3]. As technology has evolved, pediatric advocacy has moved online. For over 10 years, the AAP has used social media in its advocacy efforts using the hashtag #putkids1st. Given the wide-ranging nature of child health problems, pediatric advocacy inevitably confronts issues that lack socio-political consensus, e.g., immigrant rights, gun control, etc. Thus, while advocacy is a key pillar of the AAP and, for example, is included in mandatory training programs, others in the medical profession argue that advocacy should not be part of their essential professional commitments [6]. Notwithstanding this debate, we argue that public health advocacy that seeks to influence politicians, legislators, and voters is a form of political word of mouth, albeit more indirect. The source and message characteristics of public health advocacy by a professional association such as AAP are different than those of political parties and politicians. There is also a dearth of knowledge about what factors contribute to the use of social media as information exchanges related to health topics and for online public health advocacy. Therefore, we set out to understand the drivers of engagement outcomes by health professionals so a suitable public policy response can be formed.

Our key research question is: What source and message characteristics are associated with higher electronic word of mouth and therefore with the use of social media as a platform for advocating for public health concerns? The objective of this study is to improve our understanding of how Twitter is used for public health advocacy by the AAP and its members, and, in particular, (i) the structural dynamics of advocacy hashtag communities, (ii) the role of homophily in advocacy hashtag communities, and (iii) the antecedents of engagement with public health advocacy messages. This study presents the results of a retrospective exploratory data analysis of 4321 English language tweets generated by 1231 unique users in the calendar year 2018 featuring the hashtag #putkids1st, a hashtag community largely driven by the AAP and its members. The study makes a number of contributions to theory and practice.

## 2. Materials and Methods

### 2.1. Hashtags and Advocacy

Research suggests that social media is changing the organization dynamics and traditional forms of collective action [7,8]. Bennett and Segerberg [7] suggest that large-scale sustained movements are largely decentralized movements that combine two logics, collective action and connective action. While the former is associated with high levels of organizational resources and the formation of collective identities, the latter is based on person-to-person content sharing across media networks. Hashtags play an important role in connecting organizations and individuals to a particular movement, but also connecting movements [8]. In this way, hashtags enable both collective framing and personal framing.

As Jackson et al. [9] (p. xxix) point out, “the narrative that emerge around Twitter hashtags evolve more quickly than traditional media and, for this reason, Twitter has become one of the major tools for disseminating information to the public in the hope of spurring particular actions or outcomes”. Hashtags in social media campaigns conducted by formal and informal organizations often contain slogans. The term “slogan” has Gaelic roots, taken from “slugh gairm” meaning “battle cry”. Therefore, hashtags containing well-designed slogans have the effect of unifying and rallying support with a simple, powerfully stated message. #putkids1st is an example. Prior research has found mixed results regarding when slogans are most persuasive. McQuarrie and Mick [10] and Dass et al. [11] found that slogans have more appeal to people under low-involvement (vs. high) conditions. However, a recent empirical study found that slogans significantly increase the persuasiveness of a high involvement message among people who already agree with a position [12].

Hashtags are widely used on Twitter to discuss topics of shared interest and to discover those with similar or opposing views [13,14]. Like slogans, hashtags communicate complex messages into compact phrases while at the same time serving as an indexing mechanism for related tweets [15]. Hashtags have been widely used in the context of advocacy [16] and health communications [14]. They have been used by advocacy organizations for a variety of purposes including public knowledge and education, promoting events, mobilizing audiences via a call to action, branding, representing and communicating values and goals, to foster dialogue, to denote a time or place important to the tweet and organization, to signal a topic of interest to a specific sector or audience, or a combination of one or more of these purposes [16]. In many ways, hashtags are reminiscent of slogans due to their repeated use [15] but also in their potential, in the original etymological sense of the word slogan, to act as a 21st century battle cry.

### 2.2. Antecedents of Engagement

Social media has provided the global population with a platform to consume, produce, and share content with significantly greater reach than ever before. While enabling users to connect with each other and with content (through hashtags), it also provides users with information on content producers (bios), as well as highly visible social signals representing popularity (e.g., follower counts) and endorsement (e.g., likes, comments, and shares) [17]. The sharing of such user generated content via the Internet is often referred to as electronic word of mouth (eWOM), a term primarily associated with consumer marketing [18,19,20,21]. Due to perceptions that eWOM is perceived as more credible and relevant by consumers, it has become a common channel for political marketing. In this context, voters, candidates, and parties are often considered synonymous with consumers, products, and brands [22]. Those who participate in public advocacy efforts wish to leverage the reach that social media offers. This being the case, the antecedents of eWOM may equally apply to advocacy messaging engagement on social media.

#### 2.2.1. Source Characteristics

The antecedents of eWOM have been explored extensively in the marketing literature, including the role of source credibility [23,24,25,26,27], social reach [25], social factors, and influence [27,28,29,30], among others. In a study of antecedents of political eWOM in the Mexican presidential election, Gourinovitch et al. [31] found that user visibility, user activity, verified status, account type (i.e., party v. candidate), and social reach (i.e., number of followers) were significant factors in predicting both retweets and replies. Their findings on social reach were consistent with extant research [32,33] although both Bode and Dalrymple [32] and Walker et al. [33] found that motivation is also an important predictor of retweeting. It is important to note that the majority of research on the antecedents of political eWOM focuses on election campaigns and influencing voters. Indeed, while there is a significant literature base on the use of Twitter for social movements [34,35,36,37], there is a dearth of research on source characteristics as antecedents of advocacy eWOM, and advocacy by professional associations, such as AAP in particular.

Enli [38] suggests that the authenticity of mediated communications, such as Twitter, comprises trustworthiness, originality, and spontaneity. Indeed, authenticity has been viewed as the direct opposite of strategic self-promotion [39]—a characteristic of political social media usage.

#### 2.2.2. Message Characteristics

Like source characteristics, message characteristics are another substantial focus of research in the eWOM and advocacy eWOM literatures. These include the topic of the content, the focus of the content, structural elements (e.g., URLs, hashtags, the number of mentions, multimedia etc.), and the emotional tone of the content (sentiment) [31]. While there is an emerging literature on the role of message characteristics in eWOM, the focus of research has been typically topic categorization [40,41]. Walker et al. [33] examined the antecedents of retweeting in the 2015 U.S. general election. Their findings suggest that the sentiment and nature of appeal (e.g., attack or support) are strong antecedents of retweeting, while structural elements are weak predictors. While the findings on sentiment, in particular, are consistent with other research [42,43,44], Gourinovitch et al. [31] noted differences in the antecedents of retweeting and other eWOM behaviors, e.g., replies. They found message sentiment and hashtags are positively related to retweeting while sentiment, URLs, and mentions are negatively related to replies [31]. They also found that topics, by and large, did not have significant impact on eWOM except in two cases in which it had a negative impact, i.e., justice and environment [31]. The unique contribution of this study is to add empirical research that examines message characteristics as antecedents of eWOM for social movements and advocacy by professional associations.

The impact of the language used in Twitter messaging on eWOM has been explored from a variety of theoretic lenses included shared reality theory and regulatory mode theory. Shared reality theory suggests that humans are strongly motivated to share their understanding of the world, both in general and socially [45]. Echteroff et al. [46] suggest that shared reality involves (i) (subjectively perceived) commonality of individuals’ inner states (not just observable behaviors); (ii) a focus on some target referent; (iii) that for a shared reality to occur, the commonality of inner states must be appropriately motivated; and (iv) that shared reality involves the experience of a successful connection to other people’s inner states. Studies on shared reality theory emphasize the effect of saying-is-believing, using communication to create shared realities and enhanced recall, and specifically tuning an audience toward and about a target [47,48]—in the case of #putkids1st, children. By communicating a shared reality, people allow others to influence their judgments, and potentially their actions [45,46,49]. Regulatory Mode Theory suggests that individuals have distinct preferences for the strategies they use to pursue their goals (e.g., assessment or locomotion) [50]. While the former involves the assessment of the right action to take, locomotion emphasizes the initiation of action to achieve a goal [50]. Recent research suggests differences in goal pursuit language across the US political spectrum [51]. Consequently, Crow et al. [51] argue that the choice of such language in social media messaging constitutes a potentially powerful tool for use by policy makers in successfully conducting policy debates. They find, for example, that tweets by the Republican Party are more likely to be predominant in the language of assessment and that these types of tweets lead to more retweets [51].

### 2.3. Data and Methodology

This study investigates the antecedents of eWOM in the context of public health discussion on Twitter by leveraging a dataset of 4322 tweets (1790 original tweets, 226 replies, and 2306 retweets) mentioning the hashtag #putkids1st as generated by 1231 unique users during the calendar year of 2018. The dataset was gathered using the GNIP Historical Powertrack API which provides access to the entire Twitter firehose and, therefore, avoids sampling issues that may arise when using free historical Twitter API [52]. From the initial dataset, we extracted only original posts for the eWOM analysis as they are the ones responsible for triggering interactions, such as replies and retweets which we use as proxies for eWOM [31,33,53]. Following Walker et al. [33] and Gourinovitch et al. [31], our model includes two groups of explanatory variables, i.e., source characteristics and message characteristics.

The source characteristics considered in this study are: (i) verified status which denotes whether a user’s identity has been verified by Twitter or not1; (ii) social reach as proxied by a user’s number of followers at the time of tweeting; (iii) activity estimated as the total number of tweets, retweets, and replies posted by a user in our full dataset; (iv) visibility estimated as the total number of retweets, replies and mentions received by a user in our full dataset; (v) gender as extracted from a user’s profile where possible; (vi) parental status as extracted from a user’s bio where possible; and (vii) health sector occupation.

The message characteristics included in our model are: (i) sentiment score calculated as the difference between the number of positive and negative words in the message [54]; (ii) whether a tweet contains a link (URL) to another website; (iii) whether a tweet mentions another user (i.e., @); (iv) whether a tweet was generated by an official Twitter client (e.g., “Twitter for Desktop”, “Twitter for iPhone”, etc.) in contrast to third-party messaging platforms (e.g., “Hootsuite”, “TweetDeck”, etc.). Finally, we also considered the topic mentioned in the tweet as a potential antecedent of eWOM [31].

Tweets were manually coded by two independent coders using a grounded theory approach [55]. The 29 micro-level topics were identified and grouped into seven higher level topics, namely (i) Health (429 tweets), (ii) Nutrition (21 tweets), (iii) Safety and Security (258 tweets), (iv) Caregiving (282 tweets), (v) Early Education (5 tweets), (vi) Human Rights (12 tweets), and (vii) Other (783 tweets).

All the variables mentioned above were then included in two Ordinary Least Squares (OLS) regression models that had the natural logarithm of one plus the number of replies received by a tweet and the natural logarithm of one plus the number of retweets received by a tweet. The results of the regression analysis are presented and discussed in the next section.

## 3. Results

Unsurprisingly, given that the #putkids1st hashtag is used by the AAP to promote its advocacy efforts, a significant number of the participants in the #putkids1st hashtag community were identified as health workers (n = 669), and specifically pediatricians (n = 633). This is unsurprising. In our final dataset, 52 percent of the users were identified as male, 25 percent as female. With regard to parental status, 24 percent of users were identified as fathers while another 7 percent were identified as mothers. Finally, 72 percent of users were identified as (or employed by) a health organization.

Among the seven major topics that were identified, Health (429 tweets), Safety and Security (258 tweets), and Caregiving (282 tweets) were the most prominent. However, 783 tweets (approx. 44 percent of the tweets in our final sample) fell under the category “Other” and, therefore, could not be linked to a specific topic. Nutrition (21 tweets), Human Rights (12 tweets), and Early Education (5 tweets) only featured on a limited number of tweets, and within these three topics, access to healthcare, keeping families together, and gun control were significant topics of discourse.

Table 1 presents the results of the regression analysis. The results suggest that the only significant factor that had an impact on the number of replies received by a tweet is the Early Education topic. Unsurprisingly, the R-Squared is very low 0.024, suggesting that the model can only explain the 2 percent of the variation in the number of replies. These results may be partly due to the low number of replies (226) and, as such, there is not enough variance in the dependent variable.

The results are more interesting when we regress different source and message characteristics on the number of retweets. The results suggest that user activity and visibility is negatively associated with the number of retweets received. Similarly, users whose gender can be inferred from their profile (either male or female) tend to receive more retweets than users who cannot be classified as either male or female. Organizations, for example, would fall under this category. With regard to parental status, users who can be classified as fathers tend to receive less retweets than users whose parental status cannot be inferred. However, no statistical difference emerges between mothers and other users. Users that can be linked to organizations in the health sectors tend to receive more retweets than other users. Turning our attention to different message characteristics, tweets containing a URL and posted from an official Twitter client, and tweets that can be linked to the Caregiving topic tend to attract more retweets than others.

## 4. Discussion

Social networking sites can play a role as information exchanges related to health topics. The use of the #putkids1st slogan and hashtag by the AAP provides useful theoretical and practical insights for public health communicators and policymakers seeking to achieve societal consensus and bridge the political divide through audience tuning based on communications that emphasize shared realities and goal pursuit preferences. As discussed, users identified as health sector workers were more likely to engage than non-health workers. This may be explained by homophily, or a conceptually distinct but related construct, shared reality theory. Homophily refers to theory that similar individuals associate with each other more often than dissimilar individuals [56]. In this case, it may be that the data suggest a form of occupation or occupational value homophily where engagement is higher with others who work in the same domain, i.e., pediatrics, or who share similar beliefs about the importance of their work, i.e., ensuring the health and well-being of all children. For example, there is evidence of not only pediatricians but surgeons, social workers, and other health professionals within the wider hashtag community. In considering the design of advocacy campaigns, public health advocates and communicators should consider how the similarity of both occupation and occupational values can be leveraged to increase engagement, and as a consequence, reach.

Pediatricians, by definition, are concerned with the care of infants, children, and young people, a goal shared with the 63 million parents in the US alone [57]. We posit that the #putkids1st discourse is an example of pediatricians’ audiences tuning not only to other pediatricians but parents in general, thus crossing political divides. This shared reality can be reinforced by aligning with topical content at a given time. For example, the most discussed topics in the #putkids1st discourse included those related to access to healthcare for children, the healthcare of migrant children and gun control. This is unsurprising given that the focal period coincided with the Trump Administration migrant separation policy, the extension of CHIP, and a large number of school shootings. Consistent with previous research, these data support the proposition that Twitter largely reflects the political landscape of its time. It also suggests that public health communicators and advocates need also to be responsive and timely in their communications in order to leverage the attention and emotion generated by current news items at any given time. Audience tuning based on communicating shared realities with specific audiences, supported by trending topics, may prove a powerful took for public health advocacy and communication. Building on homophily and shared reality theory, these findings may also be explained by the similarities of hashtags and slogans. The #putkids1st community had very few dissenting contributors; the overwhelming majority of participants in the hashtag community agreed with the position embodied in the hashtag. Similar to slogans (Wood 2020), it may be that the persuasiveness, and consequently the engagement of a high involvement message is higher among people who already agree with a position.

The use of locomotion-based goal pursuit language in the hashtag, #putkids1st, as well as the wider #putkids1st discourse, potentially builds on audience tuning that targets parents but also those more susceptible to locomotion-based goal pursuit language. While it is unclear whether such strategies turn off those more inclined towards assessment-based language, e.g., supporters of the Republican Party in the US [51], to engage in eWOM, public health communicators and advocates should be cognizant of the potential differences in eWOM behavior associated with these messaging strategies. A twin-track strategy of targeted messaging not only building on shared realities but tuned to assessment- or locomotion-based language may prove impactful.

From a technical perspective, this study highlights important mechanical considerations for public health advocates and communicators with respect to stimulating eWOM. In the case of the #putkids1st discourse, less visible, less active, and transparent users were likely to generate greater eWOM. It highlights the impact of mobilizing grass roots advocates, particularly those whose expertise is established as in the case of pediatricians, who are likely perceived as more authentic than organizations or individuals with established media profiles.

## 5. Conclusions

In attempting to build both collective action and connective action on social media, professional organizations and their members appear to be most effective when they are considered authentic, trusted sources [38]. Our results also surprisingly reveal that users with low usage levels and visibility are also considered more authentic and receive more replies. Regarding the messages themselves, including a URL and the #putkids1st hashtag, posting from an official Twitter client, and insuring tweets address topics that are clearly linked to the broader Caregiving area are most effective.

The AAP and its members have advocated for public health for many years. As the domain of that advocacy moves into social media, we believe the results of this study can help guide future advocacy efforts. We believe there is abundant future research possibilities as scholars examine other source, message, and context factors that contribute to the effectiveness of advocacy on this very important area of health care and children well-being.

## Figures and Tables

**Table 1:** Regression results.

Variables	No. of Replies	No. of Replies
Verified User	−0.001	−0.208
	*−0.024*	*−0.186*
User Social Reach	0.000	−0.012
	*−0.001*	*−0.011*
User Activity	0.000	−0.091 ***
	*−0.001*	*−0.013*
User Visibility	0.000	−0.083 ***
	*−0.001*	*−0.017*
User Gender—Male	−0.002	0.082 *
	*−0.004*	*−0.048*
User Gender—Female	−0.001	0.119 ***
	*−0.004*	*−0.044*
User—Parent	0.000	0.003
	*−0.003*	*−0.037*
User Occupation—Health Sector	0.008	0.192 **
	*−0.006*	*−0.079*
Message—Sentiment Score	0.000	0.011
	*−0.001*	*−0.015*
Message—Contains URL	0.001	0.388 ***
	*−0.005*	*−0.044*
Message—Contains Mention	0.004	1.209
	*−0.003*	*−0.767*
Message Topic—Health	−0.002	0.034
	*−0.003*	*−0.036*
Message Topic—Nutrition	−0.002	−0.066
	*−0.011*	*−0.189*
Message Topic—Safety and Security	−0.001	−0.021
	*−0.004*	*−0.048*
Message Topic—Caregiving	0.001	0.308 ***
	*−0.003*	*−0.04*
Message Topic—Early Education	0.136 ***	0.009
	*−0.023*	*−0.272*
Message Topic—Human Rights	−0.003	0.247
	*−0.015*	*−0.151*
Message—From Twitter Client	0.005	0.262 ***
	*−0.006*	*−0.086*
Constant	0.000	−0.108
	*−0.010*	*−0.777*
Observations	1790	1790
R-squared	0.023	0.159

Notes: Standard errors in italics. *** *p* < 0.01; ** *p* < 0.05; * *p* < 0.10.

## Data Availability

Data was licensed directly from Twitter under an approved use case. As such, the dataset cannot be made publicly available by the authors.

## Abbreviations

The following abbreviations are used in this manuscript:

AAP	American Academy of Pediatrics
eWOM	Electronic Word of Mouth

## References

[1] World Health Organisation (2020). Children: New Threats to Health. https://www.who.int/news-room/fact-sheets/detail/children-new-threats-to-health.

[2] Uchitel J., Alden E., Bhutta Z.A., Goldhagen J., Narayan A.P., Raman S., Spencer N., Wertlieb D., Wettach J., Woolfenden S. (2019). The rights of children for optimal development and nurturing care. Pediatrics.

[3] Paulson J.A. (2001). Pediatric advocacy. Pediatr. Clin. N. Am..

[4] Oberg C.N. (2003). Pediatric advocacy: Yesterday, today, and tomorrow. Pediatrics.

[5] Christoffel K.K. (2000). Public health advocacy: Process and product. Am. J. Public Health.

[6] Huddle T.S. (2011). Perspective: Medical professionalism and medical education should not involve commitments to political advocacy. Acad. Med..

[7] Bennett W.L., Segerberg A. (2012). The logic of connective action: Digital media and the personalization of contentious politics. Inf. Commun. Soc..

[8] Hopke J.E. (2015). Hashtagging politics: Transnational anti-fracking movement Twitter practices. Soc. Media+ Soc..

[9] Jackson S.J., Bailey M., Welles B.F. (2020). # HashtagActivism: Networks of Race and Gender Justice.

[10] McQuarrie E.F., Mick D.G. (1999). Visual rhetoric in advertising: Text-interpretive, experimental, and reader-response analyses. J. Consum. Res..

[11] Dass M., Kohli C., Kumar P., Thomas S. (2014). A study of the antecedents of slogan liking. J. Bus. Res..

[12] Wood C. (2020). Slogans as Brand Proverbs and their Role in Healthcare Marketing Communications: An Exploratory Study. Association for Marketing & Health Care Research 2023 Conference Proceedings.

[13] Bruns A., Burgess J. (2011). New methodologies for researching news discussion on Twitter. Proceedings of the 3rd Future of Journalism Conference 2011.

[14] Xu W.W., Chiu I.H., Chen Y., Mukherjee T. (2015). Twitter hashtags for health: Applying network and content analyses to understand the health knowledge sharing in a Twitter-based community of practice. Qual. Quant..

[15] Kuo R. (2018). Racial justice activist hashtags: Counterpublics and discourse circulation. New Media Soc..

[16] Saxton G.D., Niyirora J., Guo C., Waters R. (2015). # AdvocatingForChange: The strategic use of hashtags in social media advocacy. Adv. Soc. Work..

[17] Khamis S., Ang L., Welling R. (2017). Self-branding, ‘micro-celebrity’and the rise of social media influencers. Celebr. Stud..

[18] Hennig-Thurau T., Walsh G., Walsh G. (2003). Electronic word-of-mouth: Motives for and consequences of reading customer articulations on the Internet. Int. J. Electron. Commer..

[19] Litvin S.W., Goldsmith R.E., Pan B. (2008). Electronic word-of-mouth in hospitality and tourism management. Tour. Manag..

[20] Bronner F., De Hoog R. (2011). Vacationers and eWOM: Who posts, and why, where, and what?. J. Travel Res..

[21] Babić Rosario A., De Valck K., Sotgiu F. (2020). Conceptualizing the electronic word-of-mouth process: What we know and need to know about eWOM creation, exposure, and evaluation. J. Acad. Mark. Sci..

[22] Chowdhury T.A., Naheed S. (2020). Word of mouth communication in political marketing: Understanding and managing referrals. J. Mark. Commun..

[23] Doh S.J., Hwang J.S. (2009). How consumers evaluate eWOM (electronic word-of-mouth) messages. Cyberpsychol. Behav..

[24] Wu P.C., Wang Y.C. (2011). The influences of electronic word-of-mouth message appeal and message source credibility on brand attitude. Asia Pac. J. Mark. Logist..

[25] Castillo C., Mendoza M., Poblete B. Information credibility on twitter. Proceedings of the 20th international Conference on World Wide Web.

[26] Morris M.R., Counts S., Roseway A., Hoff A., Schwarz J. Tweeting is believing? Understanding microblog credibility perceptions. Proceedings of the ACM 2012 Conference on Computer Supported Cooperative Work.

[27] Zhang H., Liang X., Qi C. (2021). Investigating the impact of interpersonal closeness and social status on electronic word-of-mouth effectiveness. J. Bus. Res..

[28] Bakshy E., Hofman J.M., Mason W.A., Watts D.J. Everyone’s an influencer: Quantifying influence on twitter. Proceedings of the Fourth ACM International Conference on Web Search and Data Mining.

[29] Booth N., Matic J.A. (2011). Mapping and leveraging influencers in social media to shape corporate brand perceptions. Corp. Commun. Int. J..

[30] Gvili Y., Levy S. (2018). Consumer engagement with eWOM on social media: The role of social capital. Online Inf. Rev..

[31] Gourinovitch A., Rosati P., Moreno L., Lynn T. Predicting Electronic Word of Mouth: Evidence from the 2012 Mexican Presidential Election. Proceedings of the ICORIA 2019.

[32] Bode L., Dalrymple K.E. (2016). Politics in 140 characters or less: Campaign communication, network interaction, and political participation on Twitter. J. Political Mark..

[33] Walker L., Baines P.R., Dimitriu R., Macdonald E.K. (2017). Antecedents of retweeting in a (political) marketing context. Psychol. Mark..

[34] Bruns A., Highfield T., Burgess J. (2013). The Arab Spring and social media audiences: English and Arabic Twitter users and their networks. Am. Behav. Sci..

[35] Gleason B. (2013). # Occupy Wall Street: Exploring informal learning about a social movement on Twitter. Am. Behav. Sci..

[36] Siapera E., Hunt G., Lynn T. (2015). # GazaUnderAttack: Twitter, Palestine and diffused war. Inf. Commun. Soc..

[37] Zulli D. (2020). Evaluating hashtag activism: Examining the theoretical challenges and opportunities of# BlackLivesMatter. Participations.

[38] Enli G. (2015). Mediated Authenticity: How the Media Constructs Reality.

[39] Marwick A.E., Boyd D. (2011). I tweet honestly, I tweet passionately: Twitter users, context collapse, and the imagined audience. New Media Soc..

[40] Golbeck J., Grimes J.M., Rogers A. (2010). Twitter use by the US Congress. J. Am. Soc. Inf. Sci. Technol..

[41] Hemphill L., Otterbacher J., Shapiro M. What is congress doing on twitter?. Proceedings of the 2013 Conference on Computer Supported Cooperative Work.

[42] Stieglitz S., Dang-Xuan L. (2012). Political communication and influence through microblogging–An empirical analysis of sentiment in Twitter messages and retweet behavior. Proceedings of the 2012 45th Hawaii International Conference on System Sciences.

[43] Dang-Xuan L., Stieglitz S., Wladarsch J., Neuberger C. (2013). An investigation of influentials and the role of sentiment in political communication on Twitter during election periods. Inf. Commun. Soc..

[44] Kim J., Yoo J. (2012). Role of sentiment in message propagation: Reply vs. retweet behavior in political communication. Proceedings of the 2012 International Conference on Social Informatics.

[45] Sorrentino R.M., Higgins E. (1996). Handbook of Motivation and Cognition, Volume 3: The Interpersonal Context.

[46] Echterhoff G., Higgins E.T., Levine J.M. (2009). Shared reality: Experiencing commonality with others’ inner states about the world. Perspect. Psychol. Sci..

[47] Higgins E.T., Rholes W.S. (1978). “Saying is believing”: Effects of message modification on memory and liking for the person described. J. Exp. Soc. Psychol..

[48] Higgins E.T., McCann C.D., Fondacaro R. (1982). The “communication game”: Goal-directed encoding and cognitive consequences. Soc. Cogn..

[49] Ye J., Zhao L., Huang Z., Meng F. (2021). The audience-tuning effect of negative stereotypes in communication. Front. Psychol..

[50] Kruglanski A.W., Thompson E.P., Higgins E.T., Atash M., Pierro A., Shah J.Y., Spiegel S. (2000). To “do the right thing” or to “just do it”: Locomotion and assessment as distinct self-regulatory imperatives. J. Personal. Soc. Psychol..

[51] Crow K., Galande A.S., Chylinski M., Mathmann F. (2021). Power and the Tweet: How Viral Messaging Conveys Political Advantage. J. Public Policy Mark..

[52] Morstatter F., Pfeffer J., Liu H., Carley K. Is the sample good enough? comparing data from twitter’s streaming api with twitter’s firehose. Proceedings of the International AAAI Conference on Web and Social Media.

[53] Visentin M., Tuan A., Di Domenico G. (2021). Words matter: How privacy concerns and conspiracy theories spread on twitter. Psychol. Mark..

[54] Lynn T., Rosati P., Hanlon A., Tuten T.L. (2022). Social Media Research Using Big Data: Types, Techniques, and Technologies. The SAGE Handbook of Social Media Marketing.

[55] Strauss A., Corbin J.M. (1997). Grounded Theory in Practice.

[56] Lazarsfeld P.F., Merton R.K. (1954). Friendship as a social process: A substantive and methodological analysis. Freedom Control Mod. Soc..

[57] Bureau U. (2021). Census Bureau releases new estimates on America’s families and Living Arrangements. Census. Gov. Retrieved August.

